# Factors affecting the implementation of soil conservation practices among Iranian farmers

**DOI:** 10.1038/s41598-022-12541-6

**Published:** 2022-05-19

**Authors:** Moslem Savari, Masoud Yazdanpanah, Davoud Rouzaneh

**Affiliations:** Department of Agricultural Extension and Education, Agricultural Sciences and Natural Resources University of Khuzestan, Mollasani, Iran

**Keywords:** Ecology, Evolution, Ecology, Environmental sciences

## Abstract

As soil is the basis for agriculture, soil erosion is one of the major threats to food security in arid and semi-arid regions across the world. Therefore, soil conservation is an important step to increase productivity and ensure sustainability in agriculture. To implement soil conservation measures, farmers must voluntarily adopt soil conservation behaviors. Therefore, it may be important to understand the psychological and social factors that influence farmers' environmental sustainability. Thus, in this study, social cognitive theory (SCT) was used as a theoretical framework to investigate the factors influencing Iranian farmers' soil conservation behaviors (SCBs). The results showed that SCT was a successful theory in this area as it could explain 0.662 and 0.537 percent of behavioral intentions (BI) and SCBs, respectively. Moreover, the two components of self-efficacy (SE) and outcome expectancies (OE) were the strongest SCT variables that influenced SCBs. Overall, our results may provide new insights for policymakers in the agricultural sector to reduce soil erosion.

## Introduction

Today, human beings confront unprecedented challenges due to increasing demand for food and environmental sustainability^1,2^, primarily because agriculture is suffering from a variety of climatic stresses^3–6^ and on the other hand, recently increasing pressure on farmland to food production for the growing population has led to improper land use and severe ecological damage^7,8^. In conventional agriculture, tillage practices are inappropriately carried out without regard to the negative consequences on the environment to maximize production and income^9^. In contrast, soil erosion and land degradation negatively affect the economic, social, and environmental development of agriculture^10–12^. To increase productivity and maintain the sustainability of natural resources^13^, a paradigm shift in agriculture is essential by eliminating unstable elements of conventional agriculture (plowing and tillage, depletion of soil organic matter, monocultures, etc.)^14^.

There is no doubt that soil is the most important source of production for meeting basic human needs, especially food and wood^15,16^. Although soil produces food and wood, it forms so slowly that it is practically non-renewable^17^. Therefore, a wide range of sustainable farming methods has been proposed to address the problems of food security and sustainability in agriculture across all regions of the world^18^. In other words, the application of conservation agriculture methods due to its sustainable principles such as permanent ground cover, planned crop rotation, maintenance of agricultural soil structure, integrated weed management^15,19^ as an agroecological approach has been introduced and promoted worldwide to address the concerns of sustainable agriculture^13,20^^,^^21^. This agricultural system leads farmers to tend to apply crop rotations, maintain soil fertility by preserving crop residues, and perform minimum tillage, which ultimately leads to economic and sustainable production^22,23^.

A significant proportion (70–80%) of the world's agricultural land is affected by soil erosion, according to international studies^21^. Statistics and data confirm that Asia has the highest rate of soil erosion among all continents. Moreover, among all Asian countries, Iran is at the top of the list of countries suffering from soil erosion, as about 94% of its agricultural land is affected by soil degradation^15^. This country has a large desert area and its soils are not well covered. As a result, soil erosion in Iran reaches 16.6 tons per hectare annually, with an increasing trend^24^. Due to soil and climatic conditions, there is no appropriate situation for agricultural fields in this region^25^. Moreover, low rainfall, lack of soil organic matter, erosion, and poor soil quality are other factors that limit the possibilities of agricultural land in Iran^26^. Since soil erosion irreversibly damages agricultural land, it is one of the main causes of agricultural land destruction^27^. This negative impact can be considered a vital problem as it not only reduces agricultural productivity but also contributes to desertification and more poverty in rural areas^28^.

The three main factors destroying soil are climatic, soil physical properties, and management factors^29,30^, the last of which can play a significant role. This is because although farmers use conventional methods that increase the level of soil degradation^31^, there are no measures to protect the soil from erosion^32^. Moreover, growers use inputs to increase productivity, which ultimately increases soil degradation^33^. Therefore, the adoption of soil conservation technologies can be one of the most important measures to combat erosion and soil degradation^34^. The adoption of agricultural technologies is influenced by several factors, and in many cases, soil conservation measures are not used by farmers^35,36^. New soil conservation technologies can be applied through rules and regulations, financial incentives, and voluntary behavior^24^. Incentive programs and regulations are short-term solutions, while voluntary behaviors have long-term effects^25^. Applying voluntary behaviors to soil conservation requires understanding farmers' perceptions and perspectives. Scientists have also paid great attention to it^37–40^. Considering the important role farmers play in controlling soil erosion and protecting soil, it is necessary to study and recognize the cognitive and behavioral characteristics of farmers and rural communities^21^. Although most studies on the application of soil conservation technologies have focused on economic factors^41,42^, relatively little research has been conducted on the psychological factors influencing farmers' conservation behavior^24^. Researchers have found that focusing on economic factors alone cannot fully explain people's conservation behavior because people's decisions are not always driven by economic factors^43^. According to studies on the adoption of new soil conservation technologies, changing farmers' perceptions is the most important factor influencing the adoption of these technologies^20^. Consequently, we need to change farmers' behavior to adopt the technology at the farm level so that they accept voluntary behaviors^44^, because studies have shown that one of the major obstacles to the adoption of conservation agriculture at the field is to convince farmers to engage in conservation behaviors^34^. Thereby, studies on environmental psychology have received much attention in recent decades^45^. Accordingly, in the conservation behavior area, theories such as The theory of planned behavior (TPB)^46^, Technology Acceptance Model (TAM)^47^, and Protection Motivation Theory (PMT)^48^ and Social cognitive theory (SCT)^49^ have been used to create sustainable behaviors. SCT has been successfully applied in the human behavior area because it accounts for the dynamic nature of individuals' behavior^50,51^ and explains a higher degree of variance in protective behavior^52,53^. Furthermore, because the variables in this theory are good at predicting changes in behavior, it is more important than other theories^51^. However, there is no attempt to evaluate the effect of SCT on the adoption of SCBs. To fill this gap, we focused on this theory. Therefore, this study aimed at two objectives: (i) to determine the explanatory power and efficiency of SCT in explaining SCBs, (ii) to understand the determinants in the use of SCBs, and to determine the applicable strategies in this area.

## Theoretical framework

### Social cognitive theory (SCT)

**Figure 1:**
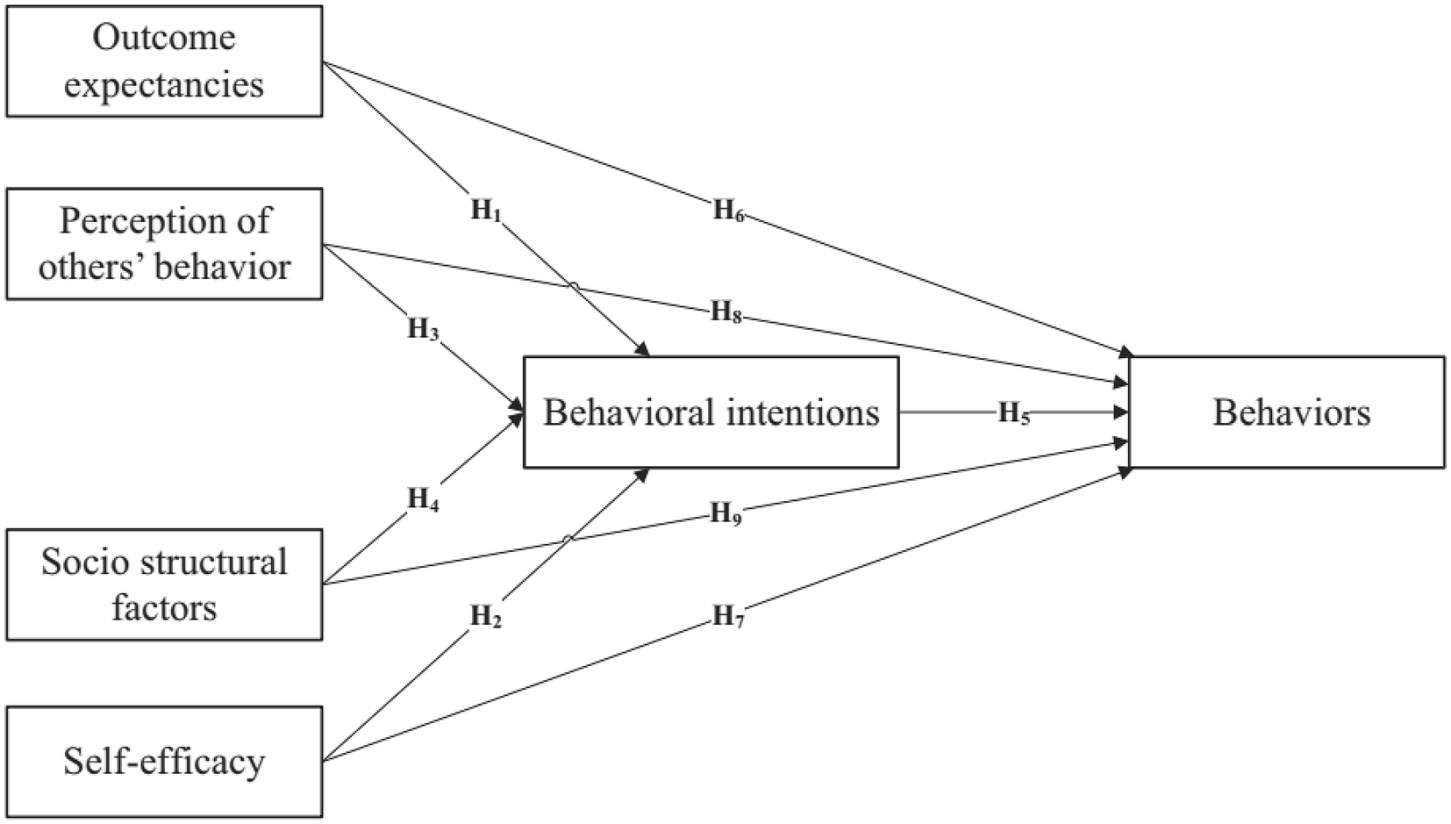
The research framework.

Social cognitive theory (SCT) was first proposed by Bandura^49^. Its application dates back to the 1970s^54^. Initially, this theory was widely used in the field of health behavior prediction and obtaining medical information systems to understand the psychological mechanisms of individuals^55^. SCT is one of the most widely used theories of behavior change, as it discusses how to establish and maintain patterns of behavior^56^. According to this theory, an individual's self-confidence plays a crucial role in his ability to perform a behavior^57^. Moreover, it assumes that human behavior is the result of a threefold reciprocal and dynamic interaction between the individual, their behavior, and the environment in which they exist^52^. Personal beliefs relate to the self-efficacy (SE) of the individual, behavioral factors include long-term goals, whereas environmental factors are obstacles and supporting factors^58^. According to this theory, behavior is influenced not only by experience but also by the observation of others^59^. Key constructs in SCT include Aim, SE, Outcome Expectancies (OE), Environmental Factors (EF), Perception of Others' Behavior (POB), and Behavioral Intentions (BI) (Bandura, 2004). SE and its reciprocal effect on the environment are key components of SCT theory^60^. Bandura^49^ describes SE as a constructive force through which human cognitive, social, emotional, and behavioral abilities are effectively organized to achieve goals. SE refers to a person's sense of empowerment and confidence to use certain behaviors to achieve a goal^57^. Situations in which people have confidence in their abilities, behavior, perceptions, and feelings are markedly different from situations in which the person feels incapable, insecure, or incompetent^61^. A strong sense of SE enhances personal well-being and ability. A person with a high SE attempts to accomplish difficult tasks and sets higher goals^60^. Conversely, people with low SE avoid challenging issues and problems. These people are weakly committed to their goals, and when faced with obstacles, they focus on their failures and negative outcomes instead of finding solutions^62^. Research shows that SE plays a significant role in influencing individual behavior and goal achievement^63^. There are two main categories of EF that influence behavior: Behavioral barriers and Social support^51,64^. Social support refers to how the behavior of others influences an individual's adoption of a behavior. Behavior change is facilitated by this factor, which provides a positive foundation for other predictors or key elements of SCT^49^. Environmental obstacles are personal and social factors that directly or indirectly hinder behavior change. The more obstacles present, the less likely people are to use behavior change techniques^65^. Facilitators and obstacles are socio-structural factors (SSFs) that are part of the environmental aspects of SCT and can predict goal attainment and behavior^66^. Thus, the environment influences the behavior of others and provides a framework for understanding behavior^49^. OE is another important construct of this theory that influences BI^59^ which can be viewed as positive and negative expectations^67^. A more positive OE provides a higher probability of adopting a particular behavior, while a lower OE is a barrier to the use of the behavior^58^. The main difference between SE and OE is that SE defines self-confidence in one's ability to perform important tasks, whereas OE defines beliefs about the consequences of such behavior^38^. Another SCT variable that directly affects behavioral choice is BI^49^, which refers to mental tendency along with desire and conscious tendency to act that is the strongest influencing variable on behavior^43,68^. Figure 1 shows the used version of SCT in this study. The research hypotheses are formed based on this version.

Layer (1): Outcome expectancies (H1), self-efficacy (H2), perception of others' behavior (H3), and socio-structural factors (H4) have significant effects on behavioral intentions.

Layer (2): Behavioral intentions (H5), outcome expectancies (H6), self-efficacy (H7), perception of others' behavior (H8), and socio-structural factors (H9) significantly affect behaviors.

## Methodology

### Study type

This practical study was a quantitative type. The data collection was conducted as a field survey that was performed as a single-cross study.

### Study area

**Figure 2:**
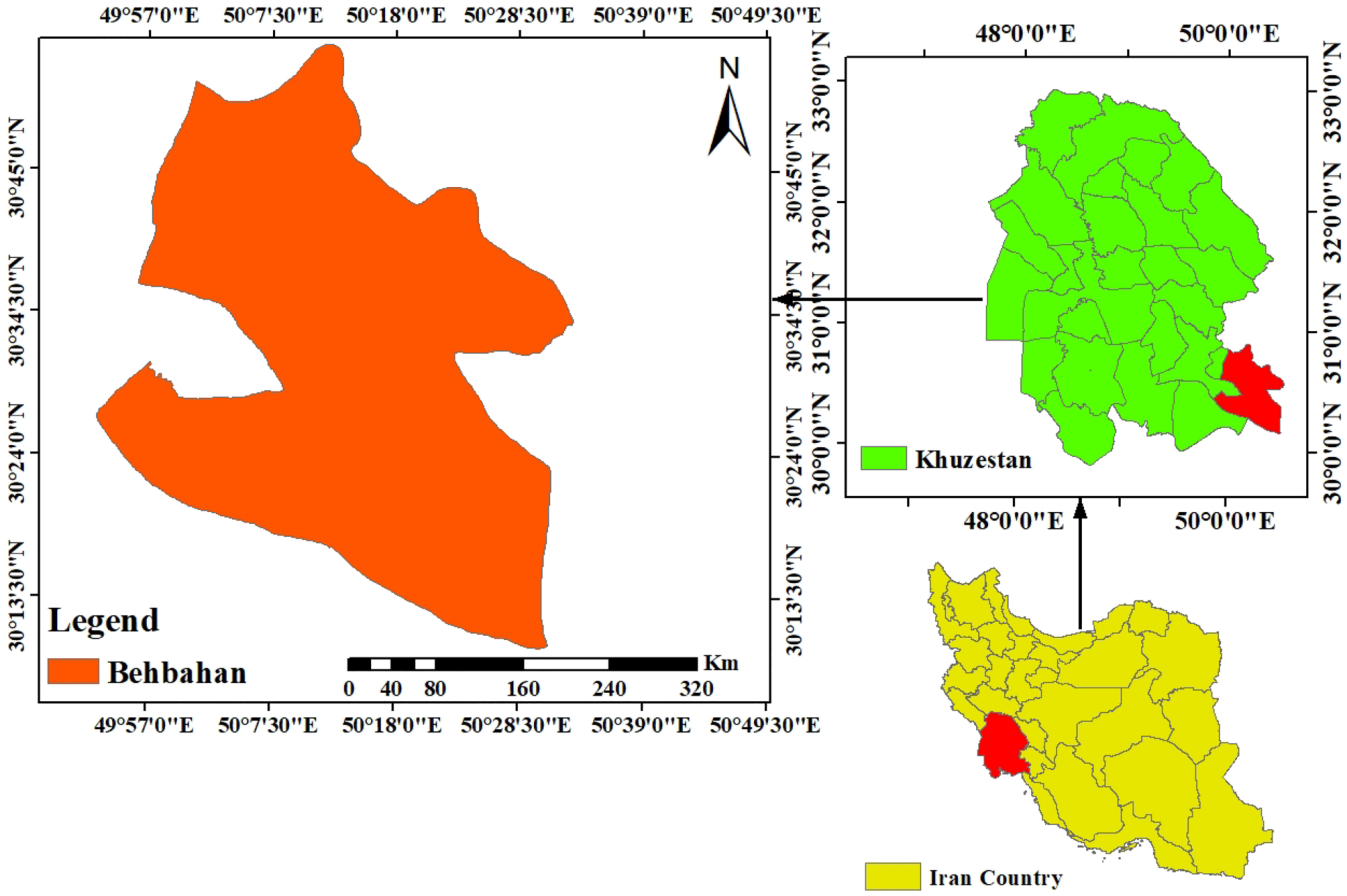
Study area.

This study was conducted in Behbahan city of Khuzestan province (southwest of Iran) (Fig. 2). The average annual precipitation and evaporation in this city are less than 255 and 2100 mm^45^, respectively. In Khuzestan province, there are about 2.3 million hectares of fertile land, of which only a small part (20%) is cultivable. On average, nearly 18 tons of soil per hectare are eroded annually^69^. Khuzestan province (including Behbahan) ranks first in Iran due to this increasing trend of soil erosion. This adverse effect caused by climatic and human factors has limited opportunities for agricultural activities^70^. In some cases, this soil degradation has even led to increased dust pollution in the region, which has ultimately affected the livability of rural households. Since climatic factors are difficult to control, they can only help reduce soil erosion by changing the behavior of farmers in an environmentally friendly way.

### Statistical population and sample size

This study included all farmers in Behbahan city, Khuzestan province. Using the table of Krejcie and Morgan, 300 participants were selected by multistage stratified random sampling method with proportional allocation. Most farmers were in the middle age group with average age of 43.68 years and they had agricultural work experience of 19.36 years. The average number of their family members was 5.16 ± 3.55. A considerable proportion of the farmers (62.7%) had attended soil conservation courses. A considerable percentage of them (37%) were also members of agricultural cooperatives. The annual income of farmers was 95.28 ± 37.53 million tomans per year (Descriptive statistics and classification of these variables are available in Table S1).

### Statement

All interviewees were informed about data protection issues by the enumerators and gave their consent orally at the beginning of each interview. Informed consent was obtained from all individual participants included in the study. All materials and methods are performed in accordance with the instructions and regulations and this research has been approved by a committee at Agricultural Sciences and Natural Resources University of Khuzestan, Mollasani, Iran. This research has been approved by a institutional review board at Agricultural Sciences and Natural Resources University of Khuzestan, Mollasani, Iran. All procedures performed in studies involving human participants were in accordance with the ethical standards of the institutional research committee and with the 1964 Helsinki declaration and its later amendments or comparable ethical standards.

### Data availability

The datasets generated during and/or analyzed during the current study are available from the corresponding author on reason able request.

### Measurements

The main instrument in this research was a questionnaire consisting of two major parts. The first one included the farmers' characteristics and their farm features. The second part consisted of items assessing SCT, which included 24 items in seven subsections: (i) four OE items (ii) three POB items (iii) three BI items (iv) three SSF items (v) four SE items, and (vi) seven items measuring SCBs. Next, the respondents were asked to comment on the statements measuring the variables as they agree with them (Likert scale 1- very low to 5 very high). The Likert scale reduces statistical problems (Fornell, 1992). One of the most important points of this research is to measure the variables SCT based on previous studies. The questionnaire items are shown in Table 1. (Descriptive statistics and classification of these variables are available in Table S2).

**Table 1:** Variables of research.

Construct	Measurement items	Sources
Behavior	Using plowing perpendicular to the field slope	Bhan and Behera^13^, Bajwa^19^, Faridi et al.^21^, Ataei et al.^24^
	Covering the soil with plant residues	
	Using animal manure on the farm	
	Using of chisel plow	
	Leveling the soil	
	No successive cultivation of a crop	
	Minimum plowing	
Self-efficacy (SE)	I'm sure I can do agricultural soil conservation operations	Bandura^49^, Yadav and Pathak^78^, Akey et al.^79^
	I have the knowledge and skills to apply soil conservation operations on my farm	
	I'm sure I can use soil conservation operations on my farm if I want to	
Socio-structural factors (SSF)	Under present conditions I must make the most of the arable land, for my income is declining	Bandura^49^, Plotnikoff et al.^66^
	Under today's busy and intellectual conditions, it is not possible for me to protect the soil on the farm	
	Under present conditions, it costs me a lot of time to protect the soil on the farm	
Behavioral intentions (BI)	I would like to use soil conservation methods soon	Kaye et al.^68^, Savari and Gharechaee^69^, Ahmmadi et al.^80^, Bagheri et al.^81^
	I plan to use soil conservation methods soon	
	I plan to use soil conservation methods soon	
Outcome expectancies (OE)	I will help prevent the destruction of our agriculture by taking soil protection measures	Bandura^49^, Shahangian et al.^51^, Thøgersen and Grønhøj^67^
	It is a great pleasure for me to participate in soil conservation efforts	
	It does not cost me much time or money to participate in conservation efforts	
	I believe that participating in soil conservation efforts is pretty smart	
Perception of others’ behavior (POB)	I believe other farmers are doing all they can to reduce soil erosion as much as possible	Bandura^49^, Shahangian et al.^51^
	The important people in my life believe that soil conservation is so important and necessary task	
	Other farmers always carry out the soil conservation behaviors and measure themselves in the field	

### Validity and reliability of the instrument

Before interviewing farmers, the draft questionnaire and questions were reviewed by a panel of experts, and based on their comments, desired changes were made to the questionnaire until it was eventually finalized. In addition, Cronbach's alpha coefficient and combined reliability were used to assess the reliability of the research instrument (Table 2).

**Table 2:** The compare of farmers' SCBs based on two-level variables.

Variable	Category	Frequency	Mean	Sd	t	Sig
Membership in cooperatives	Yes	111	2.84	0.704	4.376	0.001
	No	189	2.50	0.620		
Soil conservation courses	Yes	188	2.73	0.694	3.886	0.001
	No	112	2.44	0.592		

### Data analysis

Data were collected and analyzed using SPSS23 and SmartPls software. SmartPls was developed because of the weaknesses of first-generation structural equation modeling (SEM) and was introduced as the second generation of component-based SEM methods^71^. There are several reasons that researchers use SEM so frequently for data analysis. First, because of its ability to test theories in terms of equations between variables. Also, by considering measurement error, the researcher can analyze the data by describing the error^72^. SEM consists of two steps, measurement and a structural model^73^. Measurement is about how to explain the hidden variables by explicit variables. Namely, this step examines the validity of the explicit variables in measuring the hidden variables^74^. Structural models, on the other hand, examine the relationship between the hidden variables to test the research hypotheses^75^.

In addition, independent t-test and F-test were used to compare farmers' SCB based on two-level and multilevel variables, respectively. These two types of tests are among the parametric tests that have high accuracy for comparing the means of groups. Independent t-test is used to compare the means of two groups and F-test is used to compare the means of several groups^76^. The results of these tests have a higher validity than non-parametric tests^77^.

### Informed consent

Informed consent was obtained from all individual participants included in the study.

## Results

### Comparison of farmers SCBs based on individual and demographic variables

In order to compare farmers' SCBs based on two variables, membership in cooperatives and soil conservation courses, t-test was used. Based on the results, it can be said that farmers who were members of agricultural cooperatives or participated in soil conservation training courses had higher SCBs (Table 2).

In order to compare farmers' SCBs based on multilevel variables, age, agricultural work experience, number family members and incom, one-way ANOVA was used. Based on the results, it can be said that there was no significant difference between farmers based on these variables (Table 3).

**Table 3:** The compare of farmers' SCBs based on multilevel variables.

Variable	–	Sum of Squares	Mean Square	F	Sig
**Age**					
lower than 30	Between groups	8.368	0.349	0.828	0.699
30–50	Within groups	115.752	0.421		
More than 50	Total	124..120	–		
**Agricultural work experience**					
Lower than 15	Between groups	10.958	0.457	0.713	0.837
15–25	Within groups	176.39	0.640		
More than 25	Total	186.997	–		
**Number family members**					
Lower than 3	Between groups	9.787	0.408	0.730	0.820
3–5	Within groups	153.693	0.559		
More than 50	Total	163.480	–		
**Income**					
Lower than 75	Between groups	19.450	0.810	1.500	0.066
75–100	Within groups	148.536	0.540		
More than 100	Total	167.987			

### Structural equation modeling

In this section, the Partial Least Squares (PLS) approach was used to examine the predicted relationships in the conceptual research model. The results of this section are presented in two sections: Measurement model and research structural model.

#### Assessment of the measurement model

The assessment of the measurement model was performed in three stages: unidimensionality, Validity and Reliability, and Discriminant Validity^75,82^. The following are the results of the assessment steps for measuring research constructs.

##### Unidimensionality

This step was evaluated by the values of factor loading and t^75,82^. According to the results (Table 4), it can be assumed that this factor value for the selected markers (above 0.603) was statistically significant at the error level of (P < 0.01). This result confirms the unidimensionality of the selected markers. Consequently, the markers used to determine the research constructs were correctly selected and measured exactly the same component.

**Table 4:** Results of confirmatory factor analysis for the measurement model.

Constructs	Constructs	ƛ	t	Reliability and validity statistics
Outcome expectancies (OE)	OE1	0.847	31.224	AVE = 0.632, CR = 0.872, α = 0.804
	OE2	0.869	41.746	
	OE3	0.684	15.033	
	OE4	0.766	14.364	
Perception of others’ behavior (POB)	POB1	0.815	28.823	AVE = 0.776, CR = 0.912, α = 0.857
	POB2	0.906	65.141	
	POB3	0.918	76.032	
Behavioral intentions (BI)	BI1	0.885	54.356	AVE = , 0.803, CR = 0.926, α = 0.878
	BI2	0.893	38.964	
	BI3	0.910	53.496	
Socio-structural	SSF1	0.934	83.739	AVE = 0.761, CR = 0.905, α = 0.841
factors (SSF)	SSF2	0.774	15.091	
	SSF3	0.902	45.910	
Self-efficacy (SE)	SE1	0.757	29.870	AVE = 0.736, CR = 0.918, α = 0.880
	SE2	0.905	63.345	
	SE3	0.867	38.318	
	SE4	0.896	53.154	
Behaviors	BEH1	0.658	11.526	AVE = 0.501, CR = 0.875, α = 0.832
	BEH2	0.702	14.483	
	BEH3	0.749	21.160	
	BEH4	0.781	22.829	
	BEH5	0.603	10.206	
	BEH6	0.707	18.618	
	BEH7	0.739	18.230	

##### Validity and reliability

In this step, the values of combined reliability (CR), Cronbach's alpha, and average variance extracted (AVE) were checked^75^. As shown in Table 4, CR, Cronbach's alpha coefficient, and AVE of all constructs in the proposed research model were greater than 0.60, 0.70, and 0.50, respectively; therefore, all latent variables in the proposed research model were reliable and valid. This result indicates that the items selected to measure the research constructs are carefully chosen and allow the experiment to be repeated.

##### Discriminant validity

Diagnostic validity occurs when questions measuring one variable differ from questions measuring other variables. If the AVE between the research variables is statistically greater than the correlation between them, the research variables have adequate diagnostic validity^82^. According to Table 5, it was found that the AVE for the research constructs (0.76 < AVE < 0.89) was greater than their correlation (0.44 < r < 0.74). This result indicates that the diagnostic validity of the constructs in the proposed research model was confirmed.

**Table 5:** Correlations with square roots of the AVE.

Constructs	1	2	3	4	5	6
1. BI	0.89^a^					
2. SSF	− 0.64**	0.76^a^				
3. OE	0.63**	− 0.62**	0.79^a^			
4. POB	0.52**	− 0.53**	0.44**	0.88^a^		
5. SE	0.74**	− 0.58**	0.53**	0.47**	0.85^a^	
6. Behaviors	0.59**	− 0.45**	0.63**	0.52**	0.50**	0.87^a^

^a^The square roots of AVE estimate.

**Correlation is significant at the < 0.01 level.

#### Assessment of the research structural model

Various indicators were used in testing the fit of the structural research model (Table 6). Considering the proposed values of the indicators and the number of values given, it is obvious that the model fits well and can be used to test the research hypotheses.

**Table 6:** Summary of Goodness of Fit Indices for the Measurement Model.

Fit index	SRMR	D-G1	D-G2	NFI	rms-theta
Suggested value	< 0.1	> 0.05	> 0.05	> 0.90	≤ 0.12
Estimated value	0.08	0.775	0.452	0.98	0.08

**Figure 3:**
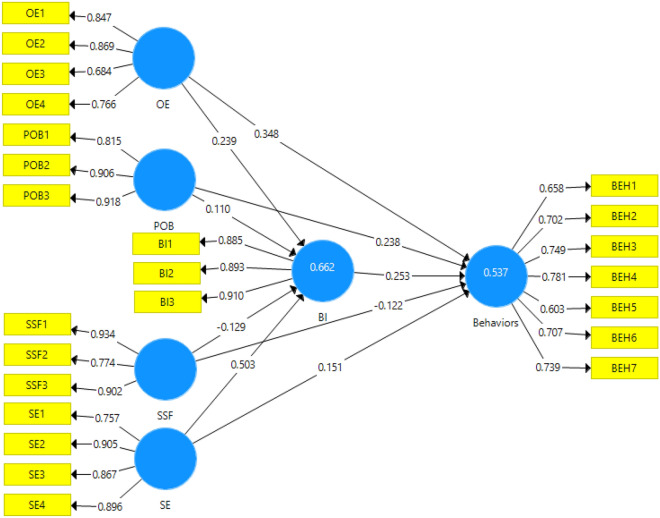
Path model with standardized factor loadings.

**Figure 4:**
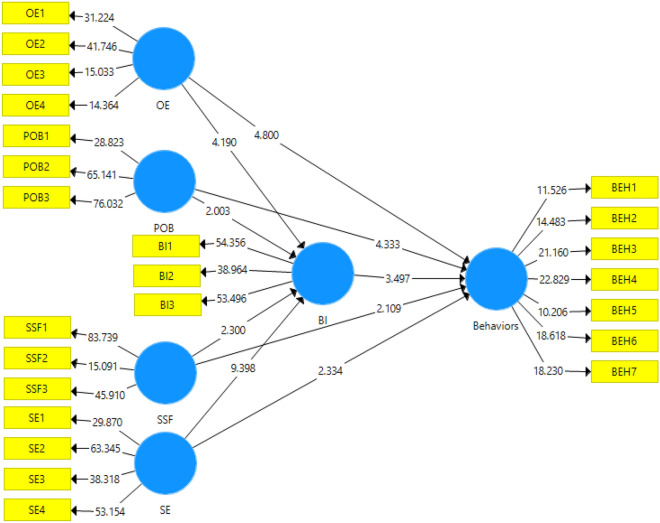
Path model with *t*-values.

After confirming the measurement and structural models of the research using confirmatory factor analysis, the method of path analysis (assessment of the structural model) was used to test the hypotheses in the proposed conceptual model of the research. The path model of the research, which shows standardized factor loadings and significance, is shown in Figs. 3 and 4.

Test of research hypotheses: this stage presents the final results of the variables related to the use of SCBs at the field level. Bootstrapping was used to assess the significance of the path coefficient or beta method. Thereby, it was used in two states, including 100 and 300 samples. The results showed that in both cases there was no change in the significance of the parameters and the results were significantly valid. Since the significance of the relationship between the variables was not affected by the sample size, while the t-statistic solely changed; therefore, hypotheses can be tested in the form of a regression model. The results showed that all the research hypotheses were confirmed based on the predicted relationships. Moreover, the research variables were able to explain 53.7% of the SCBs (Table 7).

**Table 7:** Results of research structural models.

Hypothesis	ƛ	t	Result	R^2^
H1: OE → BI	0.239	4.190	Confirm	0.662
H2: SE → BI	0.503	9.398	Confirm	
H3: POB → BI	0.110	2.003	Confirm	
H4: SSF → BI	− 0.129	2.300	Confirm	
H5: BI → behaviors	0.253	3.497	Confirm	0.537
H6: OE → behaviors	0.348	4.800	Confirm	
H7: SE → behaviors	0.151	2.334	Confirm	
H8: POB → behaviors	0.238	4.333	Confirm	
H9: SSF → behaviors	− 0.122	2.190	Confirm	

## Discussion

In this study, the decisions of Iranian farmers to use SCBs in the field were investigated using the psychological-social model of SCT. According to the authors' knowledge and literature review, there has been no attempt to study SCT among farmers around the world. While most of the studies in agriculture have studied SCT to assess water conservation behavior^51,52^ and energy use in fields^64^. Therefore, this research can fill the gap of many previous studies to some extent and provide new insights for policymakers in this area. In addition, the results of this study can assist other countries in arid and semi-arid regions of the world, as well as in tropical regions that suffer from soil erosion.

The results of comparing means showed that there is a significant difference between farmers' SCBs based on two variables; membership in cooperatives and soil conservation courses. The results of this section are in line with the studies^9,30,43^. Therefore, increasing farmers' knowledge through training courses and cooperatives has a key role in using SCBs^83^. Low levels of knowledge and skills may limit the adoption of conservation behaviors in agriculture, as the context of sustainable development activities is highly dependent on human resources^45^. In addition, the results of comparing farmers' SCBs based on the studied variables (age, agricultural work experience, the number of family members, income) showed that there was no significant difference in this area.

SEM was used to examine the factors associated with the use of SCBs in this study, and the results showed that this theory was very successful. This can be explained by two reasons: (i) All relationships between the constructs of SCT were statistically significant, and all research hypotheses were verified; therefore, it can be argued that the SCT model was much more successful in SCBs than in other application domains. (ii) SCBs have greater explanatory variance than their use in areas such as water conservation^51,52^ and renewable energy^64^. The research hypotheses are discussed below.

The SEM results showed that OE had a direct influence on BI^51,52,84–86^ and protective behavior^40^. This finding confirmed the hypotheses of 1 and 6. The reason for this result lies in the fact that a positive OE will always motivate people to continue an activity. A negative OE, on the other hand, is always seen as a major obstacle to engaging in an activity^21^. According to Bandura's^49^ theory, inhibitors and incentives can be effective in the adoption of a behavior. That is if an inappropriate behavior in the environment has positive or negative consequences for individuals, the likelihood that they will adopt the behavior changes^9,40,87^. It can be concluded that farmers who are aware of the positive consequences of using SCBs are more motivated to take action to protect the environment. According to the research of Shahangian et al.^51^ OE can take three forms: (1) a positive attitude and pleasant feeling toward participating in SCBs (expectation of a physical outcome), (2) an understanding of social support in participating in SCBs (expectation of social consequences), and (3) a sense of a moral norm in performing such behaviors (expectation of self-assessed consequences) that affect individuals' intentions and behaviors.

SE was the most influential variable on BI and SCBs^,^^9,30,57,83,87^. This result confirmed hypotheses 2 and 7. SE influences behavioral choice, effort, and goal pursuit, and determines how to deal with obstacles and challenges^60^. According to Bandura^49^ emotions, thoughts, and behavior in any situation depend on the person's sense of ability. Therefore, the use of SCBs requires good skills and knowledge so that a simple understanding of the nature of soil conservation activities influences farmers' behavior. This suggests that farmers who better understand soil conservation will be more engaged in such activities^83,86^. SE will lead farmers to believe that soil-conserving behaviors are possible. Indeed, SE creates a moral obligation among farmers to protect the soil. Therefore, it might be vital for farmers to attend seminars and workshops to become more familiar with soil conservation and understand more about the use of soil conservation measures and their effects.

According to the studies Valizadeh et al.^40^, Shahangian et al.^51^, Schunk and DiBenedett^59^, POB was also effective on BI and SCBs. Our results were consistent with hypotheses 3 and 8. Social learning through observation and imitation occurs unconsciously in many people, in both positive and negative ways, which can have effective or destructive consequences^49^. Other researchers state that the importance of observing the behavior of others influences personal behavior and believe that understanding the behavior of others is part of a person's behavior^21,59^. The significance of POB to SCBs highlights the importance of the social environment and an individual 's understanding of the consequences of others' behavior^51^. In agrarian societies, conversion is usually difficult due to financial and professional inadequacies, so they usually wait to learn the attitude and consequences of behavior from others, then accept it when there is positive feedback^7^. Similarly, Warner^88^ showed that observing the neighbors’ behavior constantly impacts further on the acceptance of a friendly behavior in the neighborhood than when a person with a high social status promotes and spreads a behavior. However, when behavior is institutionalized in society as a value or norm, violating it may lead to social exclusion for others. Therefore, farmers imitate other people to avoid social isolation and accept the behavior^43,51^. By doing so, people with higher social and economic status can influence the behaviors of farmers who are more inclined to conserve soil, since social pressure always influences the behavioral tendencies and actual behavior of individuals.

BI and SCBs were negatively affected by SSF, the fourth variable. This result was consistent with hypotheses 4 and 9. Other studies Shahangian et al.^51^, Schunk and DiBenedetto^59^, Yazdanpanah et al.^52^, Thøgersen and Grønhøj, 2010^67^, Plotnikoff et al.^66^, Burton et al.^65^ also support our findings. This result suggests that to use SCBs, the existing educational and economic barriers must be overcome. In other words, the use of soil conservation practices may initially reduce farmers' income even if it brings them long-term benefits. It can also cause a lot of wasted time for farmers, as they are not familiar with these behaviors. To solve this problem, two measures can be recommended, including (1) providing information through farmer cooperatives to familiarize farmers with a particular measure^51^, and (2) commitment of the government to cover part of the soil conservation measures cost in the early years or providing agricultural subsidies to farmers, especially to those with less financial capacity. There are numerous studies^43^ showing that increasing incentives can always improve environmental performance.

Finally, the results showed that BI positively and significantly affected farmers' SCBs, confirming hypothesis 5. According to SCT and TPB theory, psychosocial factors influencing behavior are mainly achieved through BI^46,65^. Earlier studies demonstrated that the intention variable is continuously the strongest and most direct factor influencing actual behavior^68,80,81^. According to previous studies, a possible explanation could be that people with high BI in their protective behavior, consistently perform the actual behavior more than others^45^. Therefore, policymakers in this field are generally recommended to pay special attention to the psychosocial factors mentioned in this study to create sustainable behavior, because incentives and restrictions alone cannot produce sustainable behavior in the agricultural environment (Four policy implication on SCBs among farmers are presented in text S1).

## Conclusions and limitations

This study is one of the first attempts to investigate SCBs in Iran using SCT theory. The results demonstrated that SCT was a successful theory in this area because it was able to explain 0.662 and 0.537% of BI and SCBs, respectively. Our findings could provide new insights to policymakers to increase farmers' SCBs. Moreover, according to our results, SE and OE were the strongest SCT variables in SCBs. Finally, despite the important results, three important limitations must be noted in this study. First, some variances in soil conservation behavior have not yet been explained. Therefore, it is necessary to improve the power of the model in explaining SCBs by reviewing the literature and identifying the most important variables, and including them in the SCT. Second, only SCT was used to study soil conservation behavior. Therefore, it is necessary to use other behavioral models in this area to determine their explanatory power. Third, only social and psychological factors were examined in this study. Although these factors are important components of sustainable behavior, it seems necessary to assess the economic factors outside the scope of this study.

## Supplementary Information

Supplementary Information.
