# Supplementary material for: Factors affecting the implementation of soil conservation practices among Iranian farmers
Source: Sci Rep. 2022 May 19;12:8396. doi: 10.1038/s41598-022-12541-6 (PMC9119946; doi:10.1038/s41598-022-12541-6)
Supplement: Supplementary file 1 — Supplementary Information. [file 41598_2022_12541_MOESM1_ESM.docx]

**Supplementary Information**

**Table**

**Table S1**. Demographic characteristics of farmres

| Variable | Category | Frequency | Percent | Mode |
| --- | --- | --- | --- | --- |
| Age (year) | lower than 30 | 44 | 14.7 |  |
|  | 30-50 | 170 | 56.7 | * |
|  | More than 50 | 86 | 28.6 |  |
| agricultural work experience (year) | lower than 15 | 122 | 40.7 | * |
|  | 15-25 | 105 | 35.0 |  |
|  | More than 25 | 73 | 24.3 |  |
| Number family members | lower than 3 | 56 | 18.6 |  |
|  | 3-5 | 122 | 40.7 | * |
|  | More than 50 | 122 | 40.7 |  |
| Income (million tomans) | lower than 75 | 83 | 27.7 |  |
|  | 75-100 | 132 | 44.0 | * |
|  | More than 100 | 85 | 28.3 |  |
| Soil conservation courses | Yes | 188 | 62.7 | * |
|  | NO | 112 | 112.3 |  |
| Membership in Cooperatives | Yes | 111 | 37 |  |
|  | NO | 189 | 63 | * |

**Table S2**. Grouping the situation of SCT variables among studied farmers

| Category | | | | | | SD | Mean | Variable |
| --- | --- | --- | --- | --- | --- | --- | --- | --- |
| High | | Medium | | Low | |  |  |  |
| Percent | Frequency | Percent | Frequency | Percent | Frequency |  |  |  |
| 35.7 | 107 | 37.6 | 113 | 26.7 | 80 | 0.805 | 2.30 | Outcome expectancies |
| 31.3 | 94 | 46.7 | 140 | 22.0 | 66 | 0.696 | 2.98 | Perception of others' behavior |
| 29.7 | 89 | 43.0 | 129 | 27.3 | 82 | 0.874 | 2.99 | Socio-structural factors |
| 24.3 | 73 | 39.0 | 117 | 36.7 | 110 | 0.828 | 2.61 | Self-efficacy |
| 32.7 | 98 | 45.0 | 135 | 22.3 | 67 | 0.822 | 2.36 | Behavioral intentions |
| 28.6 | 86 | 41.7 | 125 | 29.7 | 89 | 0.672 | 2.62 | Behaviors |

**Text**

**Text S1:** In general, based on the research results, the following four policies are proposed for the use of SCB among farmers. Applying these policies can affect the use of SCB among farmers.

(i) Development of farmers' professional skills: In this study, we found that farmers who participated in soil conservation training courses used SCB more than others. Therefore, it is recommended to hold courses and workshops for farmers to improve the professional skills and self-efficacy of farmers, because many of them are not aware of soil conservation methods and have always traditionally been involved in agriculture. Therefore, the use of conventional farming methods in the long run will lead to soil erosion.

(ii) Development of agricultural cooperatives: In this study, we found that farmers' membership in agricultural cooperatives has a significant effect on the use of SCB. Therefore, agricultural policy makers are advised to spontaneously form cooperatives and agricultural organizations in all rural areas, because on the one hand, these cooperatives provide knowledge sharing among farmers, and farmers will learn how to use SCB, and on the other hand will strengthen social capital among them.

(iii) Display of SCB effects: The results of this study showed that farmers' expectations of SCB will affect their behavior. It is suggested, by depicting the impact of soil erosion on the environment and farmers' livelihoods, farmers see the outcomes because outcome expectancies can encourage farmers to adopt SCBs. This is because farmers' and society's knowledge of conservation and soil erosion outcomes can not only create a positive attitude among farmers but also make SCBs a valuable norm. In addition, it is recommended that farmers be trained to use SCB at specific times through media that are accessible and usable to all farmers.

(iv) Development of moral norms: The results of this study indicated that observing the behavior of others has an effect on the use of SCB. Therefore, it is recommended, people with higher social and economic status who are more inclined to conserve soil influence the behaviors of other farmers, since social pressure always influences the behavioral tendencies and actual behavior of individuals. Therefore, when a behavior (such as SCB) is normatively institutionalized in society, it is difficult for individuals to transgress it in society because social pressures control individuals' behavior.
